# Comparison of the efficacy of thoracolumbosacral and lumbosacral orthosis for adolescent idiopathic scoliosis in patients with major thoracolumbar or lumbar curves: a prospective controlled study

**DOI:** 10.3389/fped.2024.1368201

**Published:** 2024-04-02

**Authors:** Lin Sha, Tianyuan Zhang, Wenyuan Sui, Qing Fan, Jingfan Yang, Yaolong Deng, Zifang Huang, Junlin Yang

**Affiliations:** ^1^ Department of Pediatric Orthopedics, Xinhua Hospital, Shanghai Jiao Tong University School of Medicine, Shanghai, China; ^2^ Spine Center, Xinhua Hospital, Shanghai Jiao Tong University School of Medicine, Shanghai, China; ^3^ Department of Spine Surgery, The Third Affiliated Hospital, Sun Yat-Sen University, Guangzhou, China

**Keywords:** adolescent idiopathic scoliosis, lumbosacral orthosis (LSO), brace treatment, lumbar curves, thoracolumbosacral orthosis (TLSO)

## Abstract

**Introduction:**

Thoracolumbosacral orthosis (TLSO) is the most commonly used type of brace for the conservative treatment of adolescent idiopathic scoliosis (AIS). Although lumbosacral orthosis (LSO) is designed to correct single thoracolumbar or lumbar (TL/L) curves, its effectiveness remains underexplored. This novel article aims to compare the effectiveness of LSO with TLSO in treating AIS with main TL/L curves.

**Methods:**

This prospective controlled cohort study enrolled patients with AIS with main TL/L curves and minor thoracic curves who were treated with either TLSO or LSO. Demographic and radiographic data were compared between the two groups. Treatment outcomes were also assessed. Risk factors for minor curve progression were identified, and a cut-off value was determined within the LSO group.

**Results:**

Overall, 82 patients were recruited, including 44 in the TLSO group and 38 in the LSO group. The initial TL/L curves showed no difference between both groups. However, the baseline thoracic curves were significantly larger in the TLSO group compared to the LSO group (25.98° ± 7.47° vs. 18.71° ± 5.95°, *P* < 0.001). At the last follow-up, LSO demonstrated similar effectiveness to TLSO in treating TL/L curves but was less effective for thoracic curves. The initial magnitude of thoracic curves was identified as a risk factor for minor curve outcomes in the LSO group. The ROC curve analysis determined a cut-off value of 21° for thoracic curves to predict treatment outcomes.

**Discussion:**

In contrast to TLSO, LSO exhibits comparable effectiveness in treating main TL/L curves, making it a viable clinical option; however, it is less effective for thoracic minor curves. The initial magnitude of the minor thoracic curves may guide the selection of the appropriate brace type for patients with AIS with main TL/L curves.

## 1 Introduction

Adolescent idiopathic scoliosis (AIS) is one of the most common spinal deformities in school-age children and teenagers (1). Numerous studies have validated the effectiveness of bracing for moderate curves, specifically those ranging from approximately 20° to 40°. This approach has been shown to reduce the rate of curve progression when compared to observation alone (2–4). However, many factors affect the outcomes of bracing treatment, including brace design, curve magnitude, curve type, maturity, in-brace correction (IBC), and compliance (5). Different brace designs exert varying correction forces, which influence wearing compliance and potentially affect patients' outcomes (6, 7). A more significant IBC rate and adherence to a full-time brace usually indicate a better prognosis (8, 9).

Currently, there are three types of braces according to the different anatomical regions, namely cervicothoracolumbosacral orthosis (CTLSO), thoracolumbosacral orthosis (TLSO), and lumbosacral orthosis (LSO) (10). The Milwaukee brace was a widely used CTLSO that controls upper thoracic curves in a way that traditional under-arm braces cannot (11). However, this came at the cost of impaired aesthetics and discomfort; hence, the brace was associated with poor compliance (12). The Milwaukee brace has been gradually replaced by low-profile underarm TLSO, such as Boston or Chêneau braces (13). While TLSO designs are generally more tolerated and aesthetically pleasing, compliance with these braces is not always satisfactory for some patients (14). Karol et al. reported that patients who were counselled about compliance data wore their braces for an average of 13.8 h daily, compared to 10.8 h for those who were not counselled, which were both significantly less than the recommended minimum of 20 h daily (15). Therefore, improving bracing compliance remains a significant challenge in clinical practice (16).

The LSO is primarily designed to correct single thoracolumbar or lumbar (TL/L) curves. It lacks the underarm corrective force, making it more compact, portable, and easier to wear than the TLSO (10). Theoretically, patients who wear LSO are more comfortable and have more compliance. However, only a few studies in the literature have discussed its effectiveness. According to the latest scoliosis classification of braces by the International Society On Scoliosis Orthopedic and Rehabilitation Treatment (SOSORT), the Progressive Action Short Brace (PASB) is categorized as a type of LSO, intended solely for treating thoracolumbar or lumbar (TL/L) curves. However, some earlier studies have described its original design as that of a TLSO (10, 17). Aulisa et al. reported that most patients with TL/L curves obtained curve correction after PASB treatment (18, 19). Nevertheless, they only used it for single TL/L curves, and patients with minor thoracic curves were omitted. Therefore, this novel prospective controlled study aimed to compare the effectiveness of LSO with TLSO in treating AIS with both main TL/L curves and minor thoracic curves.

## 2 Materials and methods

### 2.1 Cohorts

This prospective controlled cohort investigation was approved by the Institutional Review Board of our hospital (XHEC-C-2023-040-1) and conducted according to the principles of the Helsinki Declaration. All patients in this study visited our clinic between January 2017 and December 2021 to consult a senior physician (Prof. J.L. Yang), who has extensive expertise in the conservative treatment of AIS. The inclusion criteria were as follows: (1) AIS patients with main TL/L curves and minor thoracic curves; (2) main curve magnitude between 25° and 45°; (3) age >10 years; (4) a Risser stage of 0–2; (5) receiving TLSO or LSO with a minimum follow-up duration of 2 years; and (6) optimal bracing compliance to eliminate the potential impact of compliance on treatment outcomes. Treatment compliance was considered optimal if the difference between the prescribed bracing hours and the actual duration the brace was worn was less than 2 h (19). Compliance with bracing was assessed using patients’ self-reports and confirmed by their parents. If necessary, magnetic resonance imaging (MRI) and electromyography were performed to rule out any potential neuromuscular disorders. To enhance treatment adherence, follow-ups for patients were consistently conducted by the same doctor (Prof. J.L. Yang). Patients who had undergone other prior treatments (4 cases) or had incomplete clinical data (2 cases) were excluded from the study. Eight patients who did not reach the goals of optimal bracing compliance during treatment were also excluded. Informed consent to participate in the study was obtained from the legal guardians of the enrolled patients.

### 2.2 Bracing protocol

**Figure 1:**
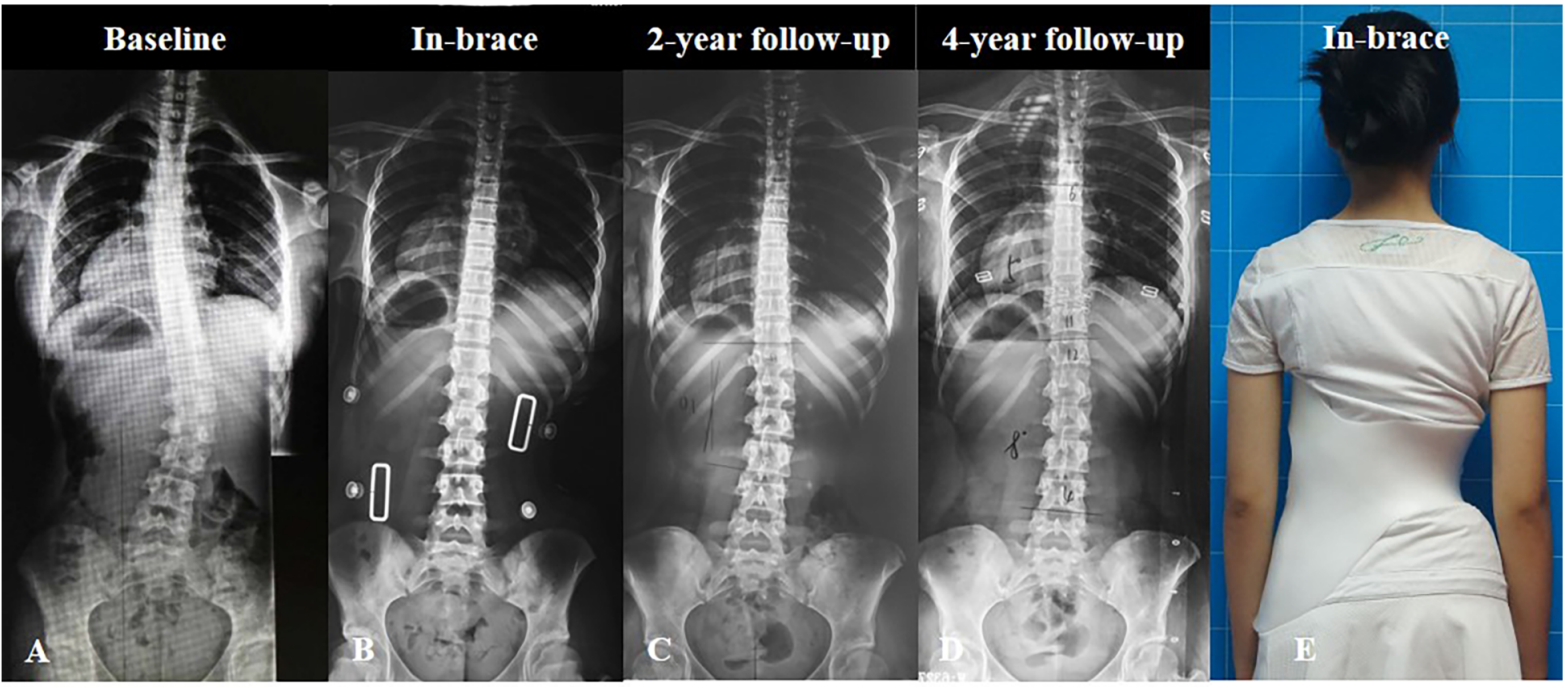
A 12-year-old girl diagnosed with adolescent idiopathic scoliosis was treated with lumbosacral orthosis (LSO). The main lumbar curve was 30° and the minor thoracic curve was 18° at baseline (**A**) The in-brace radiograph showed excellent correction of both curves (**B**) After 2 years of treatment, the patient had satisfactory curve improvement (**C**), which was adequately maintained over a 4-year follow-up period (**D**) Clinical in-brace appearance of the patient (**E**).

**Figure 2:**
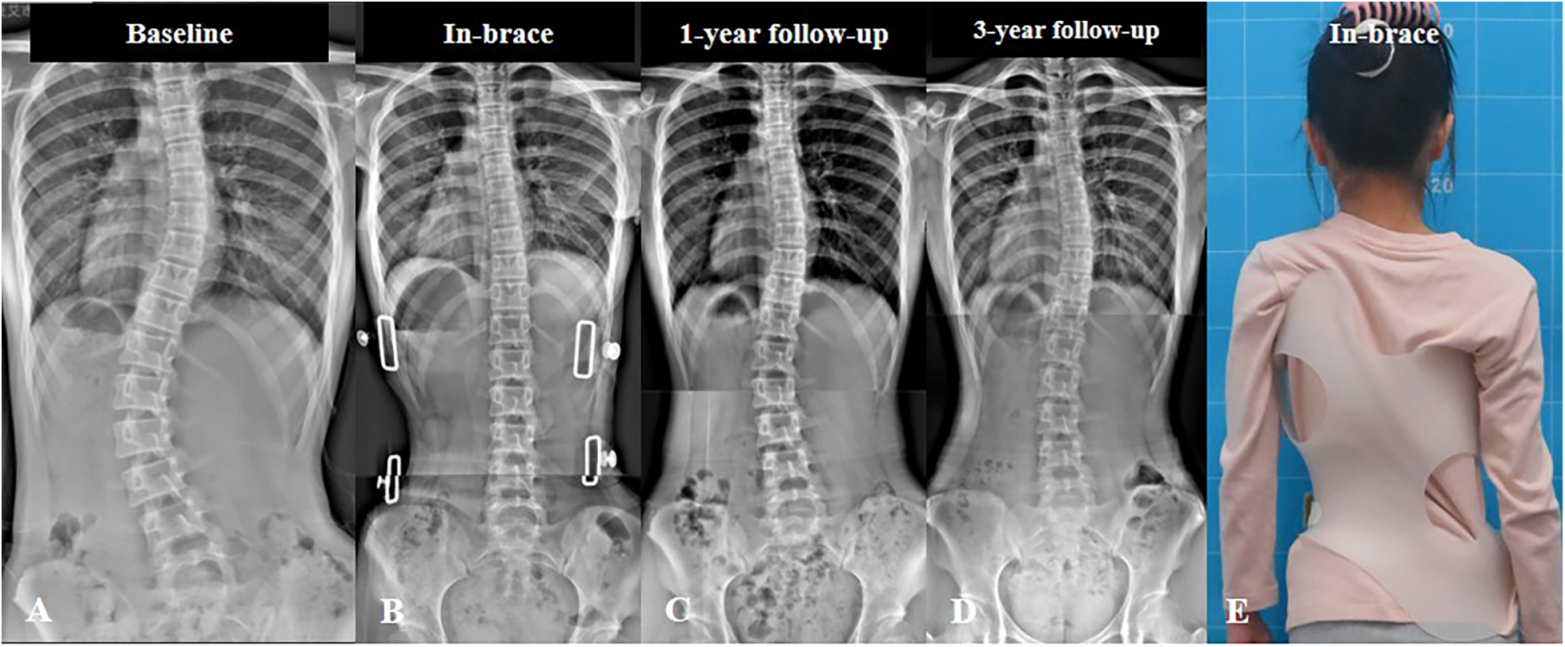
A 13-year-old girl diagnosed with adolescent idiopathic scoliosis treated with thoracolumbosacral orthosis (TLSO). The main lumbar curve was 42°, and the minor thoracic curve was 30° at baseline (**A**) In-brace radiograph shows excellent correction of both curves (**B**) After 1 year of treatment, the patient had satisfactory curve improvement (**C**), which was adequately maintained over a 3-year follow-up period (**D**) Clinical in-brace appearance of the patient (**E**).

According to the latest classification of scoliosis braces developed by SOSORT, it is necessary to delineate the following features of braces: rigidity, primary action for detorsion, primary corrective plane in three dimensions, construction type as monocot, and closure method as ventral (10). In light of the different anatomical regions where curves were controlled, both TLSO and LSO types were used in this study. The selection of the specific brace type was determined by the doctor's recommendation after fully informing the patients about the potential effects of both brace types. For minor curves ≥25°, TLSO was recommended; for minor curves <25°, LSO was used if the minor curve was considered non-structural; otherwise, a TLSO was recommended (Figures 1, 2). After the patients fit their selected braces, in-brace radiographs were taken within 2 weeks. Afterwards, regular follow-up visits were recommended every 4–6 months for all patients. The patients were also required to wear braces for at least 20 h daily. Upon reaching skeletal maturity, the patients underwent the final radiographic assessments and discontinued bracing through a weaning process.

The weaning process typically involved wearing the brace at night only, consistently for 6–12 months (20). Skeletal maturity was defined as having a Risser sign of 4 or 5, or 2 years post-menarche for female patients. The patients also underwent physiotherapeutic scoliosis-specific exercises (PSSE). It mainly comprised daily corrective postures and intensive corrective exercises, including muscle strengthening and curve stretching movements. Patients were required to exercise for at least 1 h daily (21).

### 2.3 Clinical and radiological evaluation

The demographic information collected at the initiation of brace treatment included age, sex, body mass index, and Risser sign. The patients were required to undergo full spinal posteroanterior and lateral radiographic assessments after the braces had been removed for over 24 h at each visit during follow-up. The Risser sign and curve magnitude (using Cobb's method) were measured on spinal radiographs. The main curve location was divided into TL (apex between T12 and L1) or L (apex below L1 and above L3) according to the location of the apical vertebra. The apical vertebral rotation of the main curve was assessed using the methods developed by Nash and Moe (22). The in-brace correction (IBC) rates were calculated using the following formula:

$$\begin{array}{rl} & \mathrm{In}-\mathrm{brace}\;\mathrm{correction}\;\;(\mathrm{IBC})\;\mathrm{rates}\;\\ & \;=\;(\mathrm{Cobb}\;\mathrm{angle}\;\mathrm{at}\;\mathrm{baseline}\text{-}\mathrm{in}\text{-}\mathrm{brace}\;\mathrm{Cobb}\;\mathrm{angle})\\ & \;\;/\mathrm{Cobb}\;\mathrm{angle}\;\mathrm{at}\;\mathrm{baseline}\;\;\times \;\;100\text{\%}\; \end{array}$$

The ratio of curve magnitude (minor curve/main curve) was also calculated to investigate whether the minor curve was structural or non-structural. A minor curve was considered structural if the Cobb magnitude was ≥80% of the magnitude of the primary curve (23). The Brace Questionnaire (BrQ) that explicitly evaluates the health-related quality of life of patients with AIS undergoing brace treatment was investigated for all patients in this study during follow-up (24, 25). The treatment outcome was defined based on the evolution of the main curve or the minor curve as follows: (1) improve (>5° reduction in the curve magnitude); (2) stabilize (≤5° change in the curve magnitude); and (3) progress (>5° increase in the curve magnitude). Radiographic measurements were completed by two authors (Dr. L. Sha and Dr. T.Y. Zhang), and the mean values were used in the analysis.

### 2.4 Statistical analysis

Statistical analysis was performed using the SPSS version 26.0 software (IBM Corp., Armonk, NY, USA). The univariate comparison included the independent *t*-test for comparing continuous variables and the chi-square test for comparing categorical parameters. The parameters between the two groups were compared at baseline, initial in-brace, and during the last follow-up visit. With potentially related variables entered, factors significantly associated with treatment outcome were identified by logistic regression in forward stepwise methods. The receiver operating characteristic (ROC) curve was used to determine the threshold value for treatment outcome. A *P* value of <0.05 was considered statistically significant.

## 3 Results

The study included 82 patients who met the inclusion criteria. The demographic and clinical data for these patients are presented in Table 1. Based on the type of braces used, 44 patients were fitted with TLSO and 38 with LSO. A comparison of demographic and clinical data between the two groups revealed no significant differences (Table 2, all *P* > 0.05).

**Table 1:** Demographic and clinical data of patients included in this study.

	Included patients
Numbers	82
Sex	
Female	74
Male	8
Age (years)	12.78 ± 1.31
Risser	
0	31
1	21
2	30
BMI (kg/m^2^)	17.47 ± 2.38
Initial main curve (°)	33.90 ± 6.64
Initial minor curve (°)	22.61 ± 7.68
Main curve location	
Thoracolumbar	38
Lumbar	44
Apical vertebra rotation	
1	27
2	53
3	2
Follow-up (months)	28.54 ± 4.58

**Table 2:** Comparison of demographic and clinical information between both groups.

	TLSO group	LSO group	*P* value
Numbers	44	38	–
Sex			
Female	40	34	0.827
Male	4	4	
Age (years)	12.82 ± 1.26	12.74 ± 1.37	0.780
Risser			
0	17	14	0.805
1	10	11	
2	17	13	
BMI (kg/m^2^)	17.42 ± 2.36	17.52 ± 2.43	0.864
Main curve location			
Thoracolumbar	17	21	0.131
Lumbar	27	17	
Apical vertebra rotation			
1	16	11	0.776
2	27	26	
3	1	1	
Follow-up (months)	28.41 ± 5.22	28.68 ± 3.76	0.788

In terms of radiographic evaluation, the initial main curve was 34.68° ± 6.40° in the TLSO group and 32.99° ± 6.88° in the LSO group, showing no statistically significant difference (*P* = 0.252). However, the minor curves were significantly larger in the TLSO group than in the LSO group (25.98° ± 7.47° (range, 20°–37°) vs. 18.71° ± 5.95° (range, 10°–24°), *P* < 0.001). Similarly, the minor curve/main curve ratio was also larger in the TLSO group than in the LSO group (*P* < 0.001). The IBC rates for the main curves (*P* = 0.170) were comparable between the two groups; however, the TLSO group demonstrated significantly better IBC rates for minor curves (*P* = 0.004). After an average follow-up of 28.54 ± 4.58 months, both main and minor curves showed no significant difference between the two groups (Table 3, all *P* > 0.05). Regarding health-related quality of life, patients in the LSO group had significantly higher BrQ scores than did those in the TLSO group (81.92 ± 4.19 v.s. 79.36 ± 4.40, *P* < 0.001).

**Table 3:** Comparison of radiographic parameters between both groups.

	TLSO group	LSO group	*P* value
Baseline main curve (°)	34.68 ± 6.40	32.99 ± 6.88	0.252
Baseline minor curve (°)	25.98 ± 7.47	18.71 ± 5.95	<0.001*
Minor curve/main curve (%)	74.50 ± 13.39	56.71 ± 13.47	<0.001*
In-brace main curve (°)	11.95 ± 8.29	13.11 ± 7.54	0.515
CR of main curve (%)	67.50 ± 19.94	61.39 ± 19.85	0.170
In-brace minor curve (°)	12.48 ± 7.85	11.59 ± 6.18	0.578
CR of minor curve (%)	54.32 ± 23.66	37.92 ± 26.80	0.004*
Last main curve (°)	27.02 ± 10.75	26.03 ± 10.21	0.669
Last minor curve (°)	22.64 ± 9.98	18.79 ± 8.59	0.067

CR, correction rate.

* Means significantly different.

There was no significant difference at the last follow-up for the treatment outcomes of the main curves (Table 4, *P* = 0.761). Regarding the minor curves, 21 patients had improved, 15 patients were stabilized, and 8 patients had progressed in the TLSO group. On the other hand, 7 patients had improved, 21 patients were stabilized, and 10 patients had progressed in the LSO group. The LSO group had worse treatment outcomes on minor curves than did the TLSO group (*P* = 0.017). To further investigate the factors that influence the outcomes of minor curves in the LSO group, a binary logistic regression analysis was conducted. The results showed that the initial curve magnitude of minor curves was an independent risk factor (*P* = 0.007). The ROC curve determined that a minor curve of 21° was the best cut-off value to predict the treatment outcome of minor curves. The area under the ROC curve was 0.793, with a sensitivity of 80.0% and a specificity of 82.1%, indicating good predictive capability.

**Table 4:** Comparison of treatment outcomes between both groups.

	TLSO group	LSO group	*P* value
Main curve			
Improve	30	23	0.761
Stabilize	8	9	
Progress	6	6	
Minor curve			
Improve	21	7	0.017*
Stabilize	15	21	
Progress	8	10	

* Means significantly different.

## 4 Discussion

The present study aims to evaluate whether LSO is effective in the conservative treatment of AIS in patients with main TL/L and minor thoracic curves. To the best of our knowledge, this is the first study to compare the treatment outcomes of TL/L and thoracic curves between LSO and TLSO.

We found that for the main TL/L curves, both groups showed significant improvement (both *P* < 0.001), decreasing from 34.68° ± 6.40° to 27.02° ± 10.75° and from 32.99° ± 6.88° to 26.03° ± 10.21°, respectively. The high IBC rate (averaging more than 60%) and good bracing compliance may account for the satisfactory outcomes observed. However, only patients in the TLSO group demonstrated improvement for minor thoracic curves (*P* = 0.001), decreasing from 25.98° ± 7.47° to 22.64° ± 9.98°, while the LSO group showed almost no change from 18.71° ± 5.95° to 18.79° ± 8.59° (*P* = 0.918). Moreover, the evolution of the curve magnitude also showed significantly better results in the TLSO group [47.7% (21/44) improved; 34.1% (15/44) were stabilized; and 18.2% (8/44) progressed] than in the LSO group [18.4 (7/38) improved; 55.3% (21/38) were stabilized; and 26.3% (10/38) progressed]. Therefore, the treatment outcomes of minor thoracic curves in the LSO group were worse than in the TLSO group. These results were consistent with our hypothesis. We also found that the initial thoracic curve magnitude was an independent risk factor for treatment outcomes of minor thoracic curves in the LSO group. Patients with minor thoracic curves of less than 21° may achieve better outcomes after LSO treatment based on the ROC results (Figure 1). Lastly, patients in the LSO group scored significantly higher on the BrQ than those in the TLSO group (81.92 ± 4.19 vs. 79.36 ± 4.40, *P* < 0.001), suggesting that LSO offers advantages in terms of improved quality of life and better compliance in clinical practice.

In this study, 60.5% (23/38) of patients showed TL/L curve improvement, and 15.8% (6/38) showed curve progression in the LSO group at the last follow-up. The outcomes of our study did not match the levels of curve correction seen in studies on the PASB (94% for thoracolumbar curves and 82.5% for lumbar curves). This discrepancy is likely because the initial curve magnitudes in our study were larger than in previous studies (18, 19). The mean baseline main curve was 32.99° ± 6.88° in this study, while they were 29.30° ± 5.16° for TL curves and 26.4° ± 2.8° for L curves in previous PASB studies. Furthermore, Aulisa et al. performed a long-term follow-up study of more than 10 years and found that the TL/L curves treated using PASB achieved positive long-term outcomes (26). However, minor thoracic curves were not investigated in their articles. Our study determined that a cut-off value of 21° for thoracic curves could predict the outcomes of the LSO group. Therefore, for AIS with a main TL/L curve, LSO may achieve satisfactory outcomes if the thoracic curve is less than 21°. However, if the thoracic curve is more than 21°, TLSO may achieve better control than LSO. Another potential risk of wearing LSO is the rapid progression of the thoracic curve and the need to switch to a long brace during treatment. Although some patients (10/38) in this study encountered thoracic curve progression, no patient was required to change to TLSO because the curve progressions we observed were minimal and acceptable. Moreover, the inclusion of daily physiotherapeutic exercises in the treatment regimen helped control the progression of minor curves. However, if a minor curve rapidly progresses or even exceeds the magnitude of the main curve, switching to TLSO is strongly recommended.

Besides the curve correction for thoracic curves, another difference between TLSO and LSO is patients' compliance with bracing. Theoretically, patients have better adherence to LSO than to TLSO due to its specific designs. Therefore, to eliminate the influence of bracing compliance, only patients with optimal compliance are enrolled in this study to compare the outcomes of TLSO and LSO (19). Numerous studies have identified bracing compliance as a crucial factor that influences treatment outcomes (27, 28). However, numerous factors could affect patients' adherence to braces.

Rahimi et al. concluded that compliance could be enhanced by focusing on factors related to the design and delivery of the brace. Improvements in the appearance and comfort of the brace can enhance psychological acceptance, thereby increasing compliance (29). This approach aligns with the principles of the LSO. Additionally, Karol et al. found that compliance monitoring and effective counseling could enhance patients' adherence (15). Brigham et al. reported that the most critical factors that promoted compliance with brace wearing were a patient's desire to avoid surgery and to prevent curve progression (30). Considering the above, LSO should be a possible solution for AIS with main TL/L curves in clinical practice due to its natural advantages of good compliance and better quality of life.

The main limitation of this study resides in the study design. First, the assignment of patients to the experimental group (LSO) and the control group (TLSO) was not conducted using a randomized, double-blind method. Therefore, a potential selection bias exists. Second, the total sample size of 82 patients is relatively small, which may limit the generalizability of the study's findings. A larger sample size could increase the statistical power and improve the reliability of the results. In the future, a multicenter prospective randomized controlled study of large cohorts is needed to validate the current results.

## 5 Conclusions

Compared to TLSO, LSO demonstrates similar effectiveness for main TL/L curves but is less effective for thoracic minor curves. For patients who have AIS with main TL/L and minor thoracic curves, LSO is a viable option that is associated with a better quality of life in clinical practices. The initial thoracic curve magnitude is a risk factor that affects the treatment outcomes of minor curves, and the cut-off value of 21° may guide the selection of appropriate braces.

## Data Availability

The raw data supporting the conclusions of this article will be made available by the authors, without undue reservation.

## References

[1] Weinstein SL Dolan LA Cheng JC Danielsson A Morcuende JA. Adolescent idiopathic scoliosis. Lancet. (2008) 371:1527–37. 10.1016/S0140-6736(08)60658-3 18456103

[2] Weinstein SL Dolan LA Wright JG Dobbs MB. Effects of bracing in adolescents with idiopathic scoliosis. N Engl J Med. (2013) 369:1512–21. 10.1056/NEJMoa1307337 24047455 PMC3913566

[3] Cheung JPY Cheung PWH Yeng WC Chan LCK. Does curve regression occur during underarm bracing in patients with adolescent idiopathic scoliosis? Clin Orthop Relat Res. (2020) 478:334–45. 10.1097/CORR.0000000000000989 31688210 PMC7438132

[4] Simony A Beuschau I Quisth L Jespersen SM Carreon LY Andersen MO. Providence Nighttime bracing is effective in treatment for adolescent idiopathic scoliosis even in curves larger than 35°. Eur Spine J. (2019) 28:2020–4. 10.1007/s00586-019-06077-z 31342154

[5] Hawary RE Zaaroor-Regev D Floman Y Lonner BS Alkhalife YI Betz RR. Brace treatment in adolescent idiopathic scoliosis: risk factors for failure-a literature review. Spine J. (2019) 19:1917–25. 10.1016/j.spinee.2019.07.008 31325626

[6] Li X Huo Z Hu Z Lam TP Cheng JCY Chung VC Which interventions may improve bracing compliance in adolescent idiopathic scoliosis? A systematic review and meta-analysis. PLoS One. (2022) 17:e0271612. 10.1371/journal.pone.0271612 35857763 PMC9299303

[7] Helfenstein A Lankes M Ohlert K Varoga D Hahne HJ Ulrich HW The objective determination of compliance in treatment of adolescent idiopathic scoliosis with spinal orthoses. Spine (Phila Pa 1976). (2006) 31:339–44. 10.1097/01.brs.0000197412.70050.0d 16449908

[8] Dolan LA Donzelli S Zaina F Weinstein SL Negrini S. Adolescent idiopathic scoliosis bracing success is influenced by time in brace: comparative effectiveness analysis of BrAIST and ISICO cohorts. Spine (Phila Pa 1976). (2020) 45:1193–9. 10.1097/BRS.0000000000003506 32205704

[9] Katz DE Herring JA Browne RH Kelly DM Birch JG. Brace wear control of curve progression in adolescent idiopathic scoliosis. J Bone Joint Surg Am. (2010) 92:1343–52. 10.2106/JBJS.I.01142 20516309

[10] Negrini S Aulisa AG Cerny P de Mauroy JC McAviney J Mills A The classification of scoliosis braces developed by SOSORT with SRS, ISPO, and POSNA and approved by ESPRM. Eur Spine J. (2022) 31:980–89. 10.1007/s00586-022-07131-z 35190896

[11] Lonstein JE Winter RB. The milwaukee brace for the treatment of adolescent idiopathic scoliosis. A review of one thousand and twenty patients. J Bone Joint Surg Am. (1994) 76:1207–21. 10.2106/00004623-199408000-00011 8056801

[12] Carr WA Moe JH Winter RB Lonstein JE. Treatment of idiopathic scoliosis in the Milwaukee brace. J Bone Joint Surg Am. (1980) 62:599–612. 10.2106/00004623-198062040-00015 7380859

[13] Fayssoux RS Cho RH Herman MJ. A history of bracing for idiopathic scoliosis in North America. Clin Orthop Relat Res. (2010) 468:654–64. 10.1007/s11999-009-0888-5 19462214 PMC2816759

[14] Benish BM Smith KJ Schwartz MH. Validation of a miniature thermochron for monitoring thoracolumbosacral orthosis wear time. Spine (Phila Pa 1976). (2012) 37:309–15. 10.1097/BRS.0b013e31821e1488 21540779

[15] Karol LA Virostek D Felton K Wheeler L. Effect of compliance counseling on brace use and success in patients with adolescent idiopathic scoliosis. J Bone Joint Surg Am. (2016) 98:9–14. 10.2106/JBJS.O.00359 26738898

[16] Zeck EJ Glahn Castille ME. Clinician-led mental health conversations significantly associated with outcomes for scoliosis patients. Eur J Phys Rehabil Med. (2023) 59:522–8. 10.23736/S1973-9087.23.08084-X 37746784 PMC10548397

[17] Aulisa AG Mastantuoni G Laineri M Falciglia F Giordano M Marzetti E Brace technology thematic series: the progressive action short brace (PASB), brace technology thematic series. Scoliosis. (2012) 7:6. 10.1186/1748-7161-7-6 22361349 PMC3348014

[18] Aulisa AG Guzzanti V Galli M Perisano C Falciglia F Aulisa L. Treatment of thoraco-lumbar curves in adolescent females affected by idiopathic scoliosis with a progressive action short brace (PASB): assessment of results according to the SRS committee on bracing and nonoperative management standardization criteria. Scoliosis. (2009) 4:21. 10.1186/1748-7161-4-21 19765288 PMC2754424

[19] Aulisa AG Guzzanti V Perisano C Marzetti E Falciglia F Aulisa L. Treatment of lumbar curves in scoliotic adolescent females with progressive action short brace: a case series based on the scoliosis research society committee criteria. Spine (Phila Pa 1976). (2012) 37:E786–91. 10.1097/BRS.0b013e31824b547d 22281476

[20] Wiley JW Thomson JD Mitchell TM Smith BG Banta JV. Effectiveness of the Boston brace in treatment of large curves in adolescent idiopathic scoliosis. Spine (Phila Pa 1976). (2000) 25:2326–32. 10.1097/00007632-200009150-00010 10984784

[21] Liu D Yang Y Yu X Yang J Xuan X Yang J Effects of specific exercise therapy on adolescent patients with idiopathic scoliosis: a prospective controlled cohort study. Spine (Phila Pa 1976). (2020) 45:1039–46. 10.1097/BRS.0000000000003451 32675606 PMC7373466

[22] Nash CL Jr Moe JH. A study of vertebral rotation. J Bone Joint Surg Am (1969) 51:223–9. 10.2106/00004623-196951020-00002 5767315

[23] Thompson RM Hubbard EW Jo CH Virostek D Karol LA. Brace success is related to curve type in patients with adolescent idiopathic scoliosis. J Bone Joint Surg Am. (2017) 99:923–8. 10.2106/JBJS.16.01050 28590377

[24] Liu S Zhou G Xu N Mai S Wang Q Zeng L Translation and validation of the Chinese version of brace questionnaire (BrQ). Transl Pediatr. (2021) 10:598–603. 10.21037/tp-20-377 33850818 PMC8039779

[25] Negrini S Zaina F Buyukaslan A Fortin C Karavidas N Kotwicki T Cross-cultural validation of the Italian spine youth quality of life questionnaire: the ISYQOL international. Eur J Phys Rehabil Med. (2023) 59:364–76. 10.23736/S1973-9087.23.07586-X 37195649 PMC10272934

[26] Aulisa AG Toniolo RM Falciglia F Giordano M Aulisa L. Long-term results after brace treatment with progressive action short brace in adolescent idiopathic scoliosis. Eur J Phys Rehabil Med. (2021) 57:406–13. 10.23736/S1973-9087.20.06129-8 32990686

[27] Steen H Pripp AH Lange JE Brox JI. Predictors for long-term curve progression after Boston brace treatment of idiopathic scoliosis. Eur J Phys Rehabil Med. (2021) 57:101–9. 10.23736/S1973-9087.20.06190-0 33016064

[28] Zhang T Huang Z Sui W Wei W Shao X Deng Y Intensive bracing management combined with physiotherapeutic scoliosis-specific exercises for adolescent idiopathic scoliosis patients with a major curve ranging from 40 to 60° who refused surgery: a prospective cohort study. Eur J Phys Rehabil Med. (2023) 59:212–21. 10.23736/S1973-9087.23.07605-0 36700244 PMC10167701

[29] Rahimi S Kiaghadi A Fallahian N. Effective factors on brace compliance in idiopathic scoliosis: a literature review. Disabil Rehabil Assist Technol. (2020) 15:917–23. 10.1080/17483107.2019.1629117 31248292

[30] Brigham EM Armstrong DG. Motivations for compliance with bracing in adolescent idiopathic scoliosis. Spine Deform. (2017) 5:46–51. 10.1016/j.jspd.2016.09.004 28038693

